# The Year Leading to a Supereruption

**DOI:** 10.1371/journal.pone.0159200

**Published:** 2016-07-20

**Authors:** Guilherme A. R. Gualda, Stephen R. Sutton

**Affiliations:** 1 Vanderbilt University, Earth & Environmental Sciences, PMB 351805, Nashville, TN, 37235, United States of America; 2 University of Chicago, Department of Geophysical Sciences and Center for Advanced Radiation Sources, Chicago, IL, 60637, United States of America; Heidelberg University, GERMANY

## Abstract

Supereruptions catastrophically eject 100s-1000s of km^3^ of magma to the surface in a matter of days to a few months. In this study, we use zoning in quartz crystals from the Bishop Tuff (California) to assess the timescales over which a giant magma body transitions from relatively quiescent, pre-eruptive crystallization to rapid decompression and eruption. Quartz crystals in the Bishop Tuff have distinctive rims (<200 μm thick), which are Ti-rich and bright in cathodoluminescence (CL) images, and which can be used to calculate Ti diffusional relaxation times. We use synchrotron-based x-ray microfluorescence to obtain quantitative Ti maps and profiles along rim-interior contacts in quartz at resolutions of 1–5 μm in each linear dimension. We perform CL imaging on a scanning electron microscope (SEM) using a low-energy (5 kV) incident beam to characterize these contacts in high resolution (<1 μm in linear dimensions). Quartz growth times were determined using a 1D model for Ti diffusion, assuming initial step functions. Minimum quartz growth rates were calculated using these calculated growth times and measured rim thicknesses. Maximum rim growth times span from ~1 min to 35 years, with a median of ~4 days. More than 70% of rim growth times are less than 1 year, showing that quartz rims have mostly grown in the days to months prior to eruption. Minimum growth rates show distinct modes between 10^−8^ and 10^−10^ m/s (depending on sample), revealing very fast crystal growth rates (100s of nm to 10s of μm per day). Our data show that quartz rims grew well within a year of eruption, with most of the growth happening in the weeks or days preceding eruption. Growth took place under conditions of high supersaturation, suggesting that rim growth marks the onset of decompression and the transition from pre-eruptive to syn-eruptive conditions.

## Introduction

Supereruptions have been described as the ultimate geologic hazard [1]. During one such event, hundreds to thousands of cubic kilometers of magma are expelled to the surface in a matter of days to a few months [2, 3]. Besides significant destruction on a local to regional scale, ash dispersed in the atmosphere would remain suspended and affect the weather globally for at least a couple of years [4, 5]. The Tambora eruption in 1815 is the largest known historical eruption, having erupted a total of ~180 km^3^ in only a few days [6]. It caused what has been known as the “Year without a summer” in 1816, leading to famine, health crises, and civil unrest worldwide for much of the subsequent decade [7]. Against the potential impacts of supereruptions stands our lack of direct knowledge of the processes and signals leading to supereruptions. In many ways, understanding the potential hazards associated with supereruptions is the ultimate geologic exercise, in which we are pressed to learn as much as possible from the geologic record of past supereruptions. In this paper, we examine pumice clasts–remnants of parcels of the original magma body in the subsurface–to assess the timescales over which the transition from relatively quiescent pre-eruptive crystallization to syn-eruptive decompression and crystallization takes place.

Our focus is on the Bishop Tuff, a deposit formed from the supereruption that led to the origin of the Long Valley Caldera in central California [8–10]. The Bishop Tuff has been the target of extensive field [2, 11, 12], geochemical [8, 9, 13–21], geochronological [22–25], and textural [14, 26–32] studies, to name a few, making it one of the best-studied supereruption deposits in the world. In particular, the timing of crystallization events has received considerable attention [10, 22, 25, 28, 30, 33–36]. As such, the Bishop Tuff is an ideal target to understand the timescales associated with the transition from the pre- to syn-eruptive stages of crystallization.

It has been known for more than 20 years that quartz and sanidine crystals in Bishop Tuff pumice clasts have distinctive rims (up to ~100 μm thick) [14, 31, 37], and similar features have been identified in various deposits worldwide (e.g., [38, 39, 40]). Quartz crystal rims are Ti-rich and often appear bright in cathodoluminescence (CL) images [31], while sanidine crystals have Ba-rich rims that also appear bright in CL images [14, 37]. Rims that are bright in CL images and enriched in Ti are also observed in zircon from parts of the Bishop Tuff [20, 34]. Quartz geospeedometry performed to date has led to the conclusion that these rims have formed in the final 100 years before eruption [28, 32, 36]. Yet, these timescales represent only maximum bounds on the growth times associated with the development of these crystal rims. In this work, we use synchrotron-based x-ray microfluorescence (i.e., x-ray microprobe) to explore the correlations between Ti concentration and CL intensity in quartz; we demonstrate a direct relationship between the two across the contact between bright-CL rims and dull-CL interiors [28, 32, 38]. Given this direct correlation, we use low-energy CL imaging of quartz crystals using a scanning electron microscope (SEM) to obtain high-resolution images of the rim-interior contacts. Finally, we use intensity gradients extracted from the CL images to calculate Ti diffusional relaxation timescales [28], which provide estimates of the timescales over which the transition from pre-eruptive to syn-eruptive crystallization took place during the evolution of the Bishop Tuff magmas. We show that low-energy CL imaging allows us to constrain the sharpness of these contacts–and the timescales over which this important transition took place–in unprecedented level of temporal detail.

## Materials and Methods

### Samples studied

We studied quartz crystals from Bishop Tuff pumice clasts collected from two localities: Chalfant Quarry [26] and Aeolian Buttes [30]. Sample location information is given in these references. The samples used in this study were collected by Alfred Anderson (Gualda's PhD advisor) in 1997. Both of the sampled localities are federal land in which no permits are required for sample collection. The studied samples span a range of units from early-erupted through late-erupted (fall units F7, F8 and flow units Ig1E, Ig2Ea and Ig2NW; following the nomenclature of [2]). We separated whole crystals from each pumice clast by crushing it by lightly hitting it with a baseball bat, followed by sieving (to fractions finer than 1.8 mm), winnowing in water to separate vesicular glass from crystals, and hand-picking of quartz crystals in refractive index oil under an optical microscope [26]. Crystals were grouped according to size and mounted on circular glass slides using epoxy, and then polished down to a depth sufficient to expose as much as possible of grain interiors.

Because of the importance of rim compositions to the present study, we were careful to select for analysis crystals that after sectioning showed glass attached to the edge of the crystal, such that we are confident that the actual rim of the crystal is preserved after laboratory processing. During lab processing, many crystals have some of their edges accidentally removed; in those instances, we focused work in areas where glass is still preserved.

### Analytical methods

CL imaging was performed using a Tescan VEGA3 variable-pressure scanning electron microscope (SEM) equipped with a Tescan panchromatic CL detector installed at Vanderbilt University. We employed 5, 10, and 15 kV for the electron beam accelerating voltage to compare the effect of beam acceleration on image resolution. We imaged regions of contact between rim and interior in high resolution using a pixel size of ~0.2 μm (200 μm field of view, 1024 pixel-wide images), using dwell times per pixel of either 1 ms or 3.2 ms. Under these conditions, the pixel size is typically larger than the electron beam width and the diameter and depth of its interaction volume (both >300 nm, see below); this means that the images oversample the contacts, guaranteeing that the maximum resolution possible for a given beam energy is attained. The contacts of interest were placed in a direction perpendicular to the scanning direction in the SEM to maximize their sharpness and to avoid artifacts due to possible changes in beam intensity over time during image acquisition.

We used synchrotron-based x-ray microfluorescence to acquire quantitative compositional line profiles and 2D maps of selected areas in the contact between rim and interior. The goal was to ascertain whether the CL variations observed along such contacts were due primarily to Ti zoning. We used the x-ray microprobe at the GeoSoilEnviroCARS insertion device beamline 13IDC at the Advanced Photon Source, Argonne National Lab [41]. It uses a highly focused, synchrotron x-ray beam to generate x-ray fluorescence spectra from the sample, which are collected using an energy-dispersive spectrometer (EDS). The main advantage of this technique is the combination of very low background and high spatial resolution. The main limitation resides in the relatively low spectral resolution of the EDS detector, such that elemental interferences can be problematic [41]; however, for Ti analysis in quartz, there are no significant interferences. In addition to Ti and Si (necessary for Ti quantification), we analyzed for Ca and K, so as to be able to identify the contact between quartz and glass. Even though the incident x-ray beam can be focused to sizes <1 μm in width, scattering within the sample and secondary fluorescence effectively enlarge the size of the spot from which characteristic x-ray emission takes place–this ultimately limits the resolution attainable to 1–5 μm in each linear dimension, depending on the energy of the incident beam and of the element of interest. In our case, the x-ray microprobe yields 2σ relative uncertainty close to 2.5% for ca. 50 ppm Ti in quartz, with a <5 μm wide spot.

One relevant consideration is the relative orientation of the contact surface separating rim and contact relative to the orientation of the incident and emitted beams. If the incident beam is not parallel to the contact surface at the region of incidence, the contact will be blurred in the resulting profiles and images. The geometry of the incident beam at the x-ray microprobe (at 45° from the sample surface) and the greater depth from which x-rays are produced both conspire to make x-ray microfluorescence images and profiles more blurred than CL images (incidence and collection are normal to the sample surface in CL using the SEM).

### Diffusion chronometry

**Fig 1:**
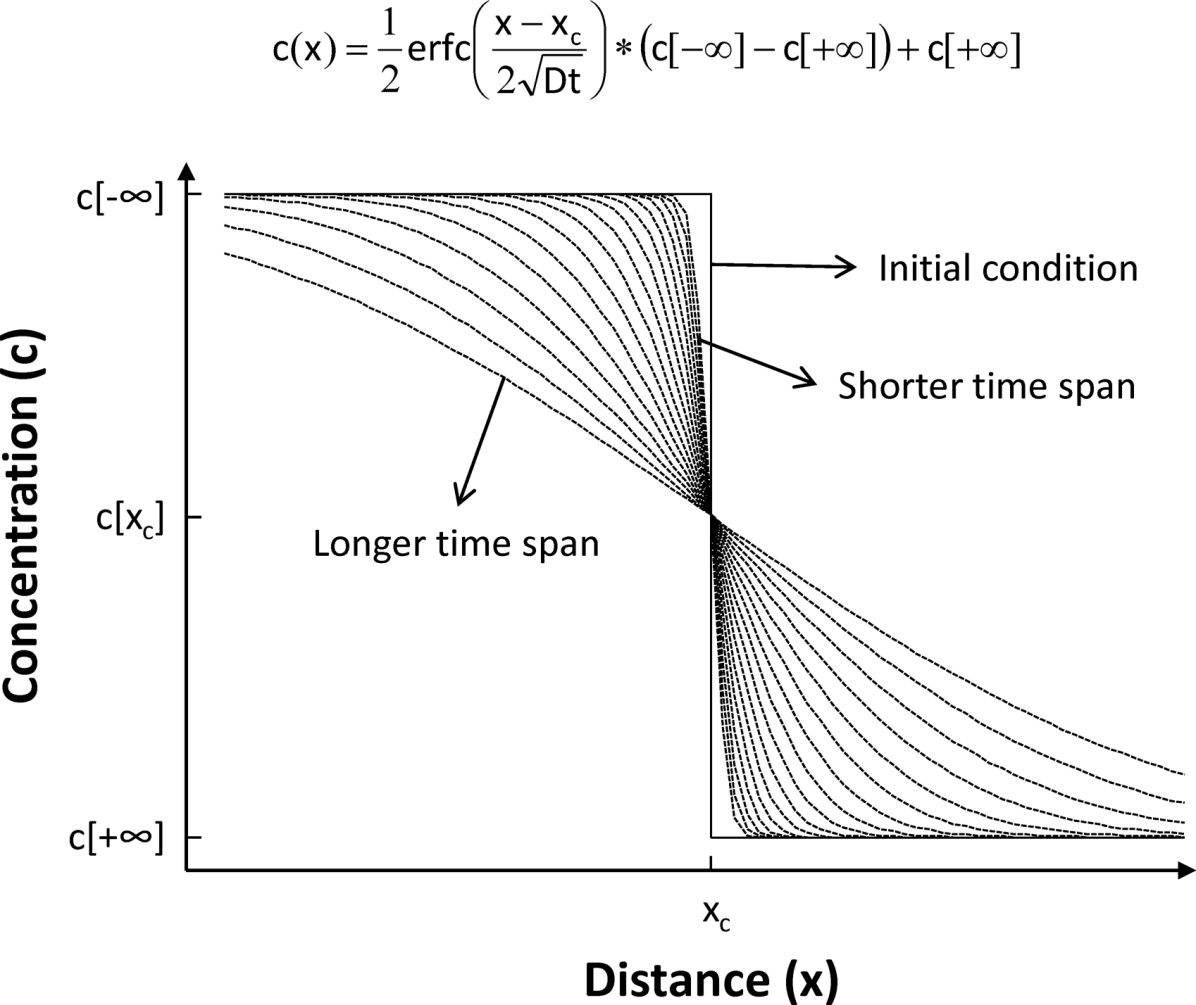
Principle behind diffusion chronometry. We use a 1D model to calculate the time since the inception of a given compositional contact in a crystal. We assume an initially infinitely sharp “step function” boundary (indicated as “Initial condition” in the diagram), which becomes progressively less sharp with time (shown as a series of curves). This problem has a well-known solution, with the resulting damped curve being described by a complementary error function (erfc) as shown in the figure. We use a least-squares procedure to find the best-fit erfc function, from which we can extract the value of ($2\sqrt{Dt}$); using experimentally determined values of the diffusion coefficient D, we can then calculate the residence time of a given contact and the growth time for the crystal region rimward from the contact. We can also measure the growth distance from our images, from which we calculate average crystal rim growth rates.

Timescales of crystallization were determined from CL profiles in quartz using a 1D model in which diffusion acts to smooth an initially sharp boundary between two semi-infinite regions of initially constant, but different, composition (see Fig 1). This diffusion problem has a well-known solution [42], in which the degree of smoothing depends on–as is typically the case in diffusion problems–the product between the diffusion coefficient (D) and time (t), and the resulting concentration (c) profile as a function of distance (x) is described by a complementary error function (erfc). We use a least-square minimization procedure to find the complementary error function that best matches each observed profile, by varying the concentration in the far field (c[-∞], c[+∞]), the center of the diffusion profile (x_c_), and the diffusion length scale ($\sqrt{\text{Dt}}$). By taking advantage of experimentally determined diffusion rates for Ti in quartz [43], we can calculate relaxation times from the fitted curves. Importantly, diffusion rates in quartz are independent of orientation [43], so that orientation of the analyzed crystals is unimportant.

Errors associated with such a fitting exercise have been discussed elsewhere [28] and suggest that uncertainties associated with calculated times are on the order of 100% (i.e., a factor of 2), which is much smaller than the grain-to-grain variability typically observed. One important contribution to the total uncertainty is the temperature of crystallization. However, while magmatic temperatures can fluctuate over the course of crystallization, rhyolitic magmas like those studied here are nearly eutectic, such that temperature variations are buffered by the crystallizing assemblage [10, 14, 28, 44, 45]. As a result, we assume a constant crystallization temperature of 750°C–consistent with zircon saturation geothermometry and rhyolite-MELTS calculations [10, 28, 44]–with an uncertainty of 30°C, so as to encompass the whole spectrum of suggested temperatures for crystallization of Bishop Tuff magmas [8, 9]; for more details, see [28].

During crystal growth, diffusion begins to relax a given contact as soon as it is established; diffusion effectively stops upon eruption, given the significant temperature decrease. Hence, the calculated time represents the time a contact spent under magmatic conditions, which also corresponds to the growth time for the region rimward from the contact [28]. We thus also measure the distance between the contact and the rim of the crystal to calculate relevant crystal growth rates for rim crystallization. The assumption of an initially infinitely sharp boundary and the potential blurring due to the orientation of the rim-interior contact surface both render the calculated times as maximum estimates, and the calculated growth rates as minimum estimates.

For each crystal rim investigated, we extracted from CL images 11 parallel profiles for each contact (5 on each side of the selected profile), and relaxation times were calculated for each of them. Given that resulting relaxation times are maximum estimates of residence time, we selected the minimum value of the 11 profiles for each contact as representing the best estimate of the crystallization time for that crystal rim. We avoid averaging multiple profiles, as the result of such averaging is inevitably to lead to smoothing of the rim-interior gradients. Instead, we also consider median values of multiple profiles, which provide very conservative estimates of rim crystallization times.

## Results

### Correlation between Ti and CL in quartz

**Fig 2:**
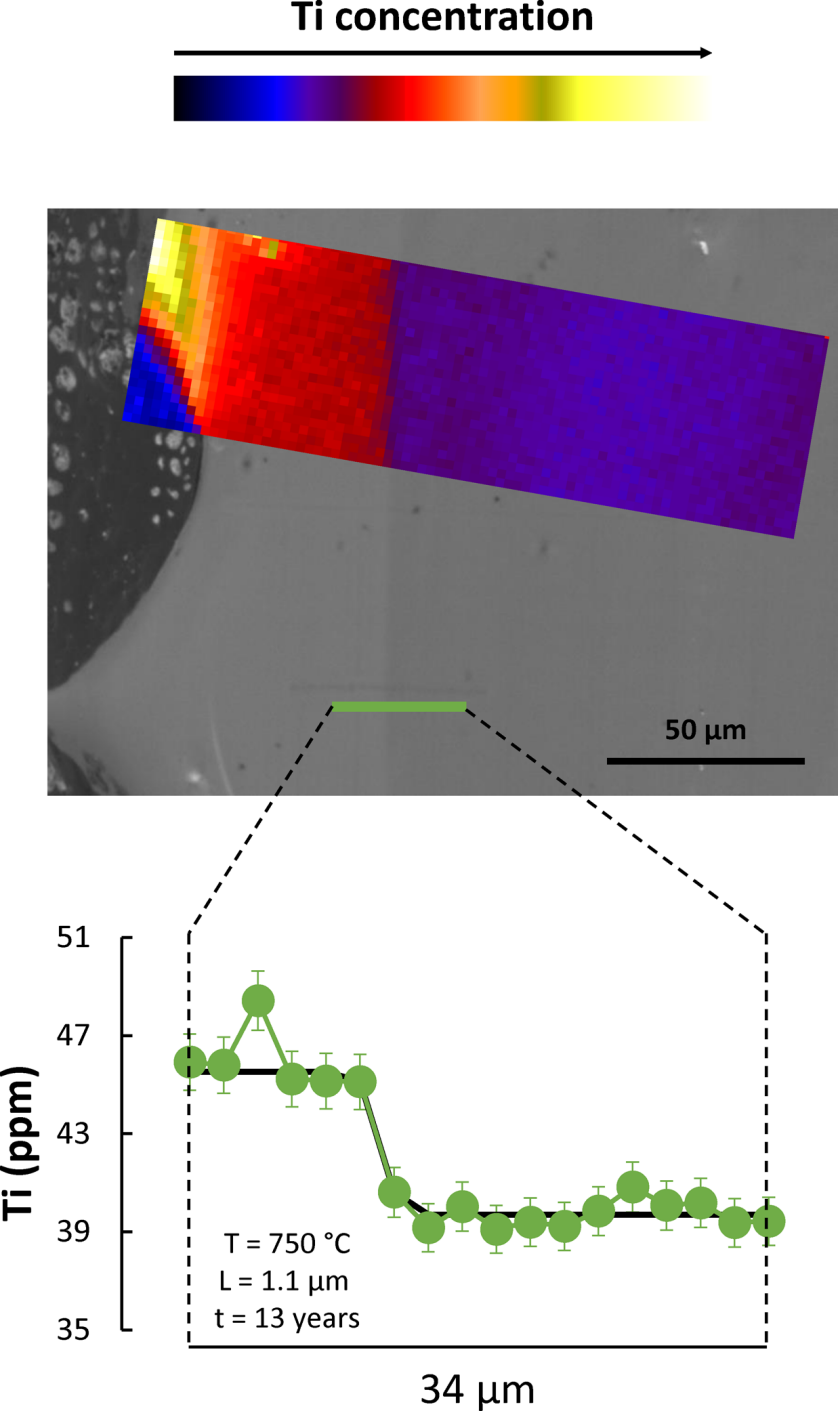
Correlation between CL intensity and Ti concentration in quartz. Central grayscale image is a detailed cathodoluminescence (CL) image of the rim region of a quartz crystal (fall unit F8 of the Bishop Tuff, following nomenclature of [2]), including the boundary between the bright-CL rim and the duller-CL interior. Color inset is a Ti map obtained using the x-ray microprobe at insertion device beamline of GeoSoilEnviroCARS (Advanced Photon Source, Argonne National Laboratory); color scale corresponds to Ti concentration, as indicated on the top bar. The correlation between CL and Ti concentration is apparent, with largely homogeneous Ti concentrations in the rim and in the interior of the crystal, and a very sharp transition between the two. The bottom diagram shows a quantitative profile across the same boundary in a slightly different position, as indicated in the CL image. The transition between bright-CL, high-Ti rim and interior takes place within one step in the profile, which corresponds to 2 μm; this shows that the diffusional length scale of interest is on the order of 1 μm or less. We obtain a maximum growth time for this contact of 13 years (fitted complementary error function shown in black), which places an upper bound on the growth time for these rims.

One of the challenges in studying quartz is its relative purity; Ti, Al, and Li are the only relatively abundant elements, reaching concentrations of tens of ppm, while other elements appear in lower concentrations. This makes detailed characterization of zoning in quartz particularly challenging. Yet, quartz is a major phase in many silicic magmatic rocks, and the ability to decode a record of zoning and crystallization in quartz can be extremely useful. It is in this context that the correlation between CL intensity and Ti concentration in quartz [28, 31, 32, 38, 39, 46] has been explored, given that CL images can be obtained much more quickly and in much higher spatial resolution then Ti maps. However, Ti is not the only CL activator in quartz, with significant CL signal deriving from other activators, particularly crystallographic defects [31]. Several datasets [28, 38, 39, 46] present data that indicate that large variations in CL intensity correlate well with variations in Ti concentration in quartz; but they also show that smaller variations in CL intensity are not explained well by Ti variations. Our results (Fig 2) confirm such conclusions, and they also lend support to the prevailing idea that Ti^4+^ is present in quartz as part of the crystal lattice, presumably substituting Si^4+^ cations [47]. Importantly for this study, our data clearly demonstrate that the contacts between rim and interior are characterized by a significant change in Ti concentration, with a transition that is as steep as can be imaged with x-ray microfluorescence. That being the case, we use CL intensity as a proxy for Ti concentration, and we use CL images (particularly, profiles extracted from such images) to characterize in detail–much greater than is possible with direct quantification of Ti concentrations–the variation in Ti across rim-interior contacts in quartz from the Bishop Tuff.

### CL imaging conditions

**Fig 3:**
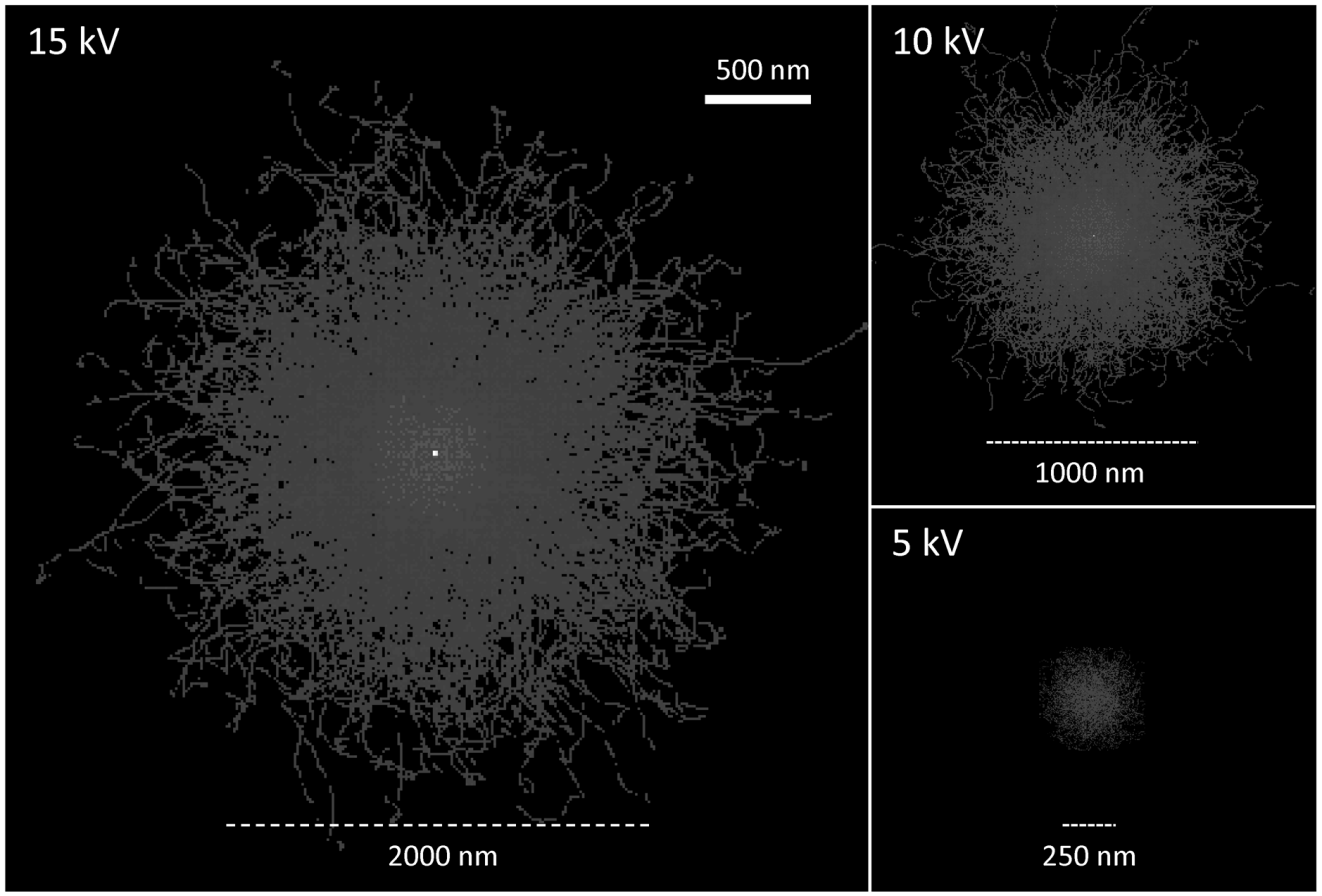
Effect of incident electron beam energy on excitation volume within a quartz crystal. Results shown are derived from Monte Simulations performed using the software MC X-Ray Lite (version 1.4.6.0, http://montecarlomodeling.mcgill.ca/software/mcxray/mcxray.html), assuming a quartz crystal with density of 2.65 g/cm^3^; calculations for 1000 electrons are shown as the depth-integrated paths on the plane perpendicular to incidence of the electron beam. Scale is the same for the three images shown. Horizontal bars are for reference; they show the approximate size of the beam at each energy. Notice the very strong effect of beam energy on the diameter of the excited volume, which translates into a strong dependency of maximum possible cathodoluminescence (CL) image resolution with beam energy.

**Fig 4:**
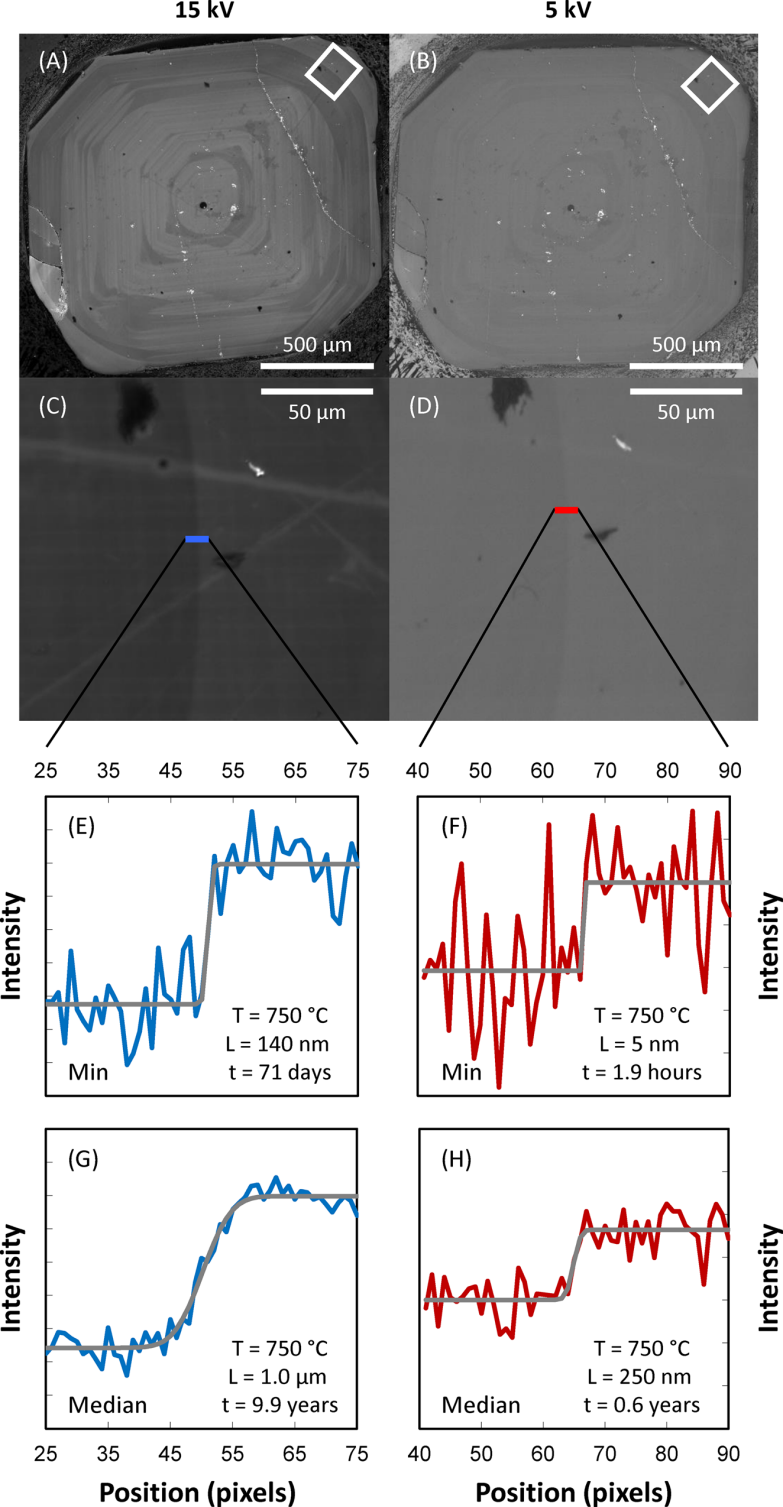
Comparison of CL images and derived intensity profiles using incident electron beam energies of 15 kV (left panels) and 5 kV (right panels). (A, C, E, G) Data obtained using 15 kV electron beam. (B, D, F, H) Data obtained using 5 kV electron beam. (A-B) Low-resolution images showing a whole quartz crystal with a distinct bright-CL rim. (C-D) High-resolution images (195 nm per pixel) of the contact between bright rim and duller interior (location within the crystal shown in A-B as white rectangle). (E-H) CL intensity profiles extracted from high-resolution images (locations shown), with best fit error functions displayed as gray curves; the length scale of diffusional relaxation (L) extracted from the fitted curves (assuming an initial step function) are indicated, as well as the derived growth times assuming a temperature of 750°C (following [10]). Estimated errors are less than a factor of 2 (see [28] for details), which, despite relatively large, do not affect any of the interpretations or conclusions. (E-F) Calculations showing profiles resulting in minimum growth times (out of 11 parallel profiles) in each case. (G-H) Calculations showing profiles consisting of median values (out of the same 11 parallel profiles) at each position in each case. Crystal is from fall unit F8 (following the nomenclature of [2]), sample F8-15.

Over the course of this study, it became clear that Ti (and, accordingly, CL) gradients across the rim-interior contacts are very steep. Inspection of the Ti zoning profiles (Fig 2) shows that gradients are steeper than what can be properly characterized with the x-ray microprobe–this reveals characteristic diffusion length scales of interest smaller than 1 μm. This poses significant challenges for adequate imaging of zoning in quartz, given that the excitation volume–the region from which signal is emitted from within a quartz crystal–can be of the same order of magnitude for typical electron beam accelerations (~1–20 kV). We take advantage of the fact that the size of the excitation volume is a strong function of the incident electron beam energy [48], such that lowering the incident beam energy results in improved CL image resolution (Fig 3). At the same time, incident energy also strongly controls the signal-to-background ratio, with the result that lower energy leads to noisier images with reduced contrast (Fig 4). As such, choosing the most appropriate beam energy for CL imaging becomes critical. We attempted imaging at 5, 10, and 15 kV to find the best compromise between image resolution and image contrast. Where possible, we employed a 5 kV beam, which results in significantly improved spatial resolution (<300 nm; see Fig 3), but results in much reduced CL contrast (Fig 4). This is adequate for imaging rim-interior contacts of crystals from pumice clasts in which rims are substantially brighter than crystal interiors (a total of 63 contacts from 3 samples). In some pumice clasts, quartz rims are less distinct and show more limited contrast between rim and interior; in these cases, imaging was performed at 15 kV (10 contacts in 2 pumice clasts), which ultimately limits the resolution of the resulting images and derived time estimates. We will focus on the 5 kV results, given that they yield better constraints on rim crystallization times, but we present results derived from 15 kV images for the two samples in which imaging at 5 kV was not practical (early-erupted samples F7-12 and F7-14).

### Rim growth times

**Fig 5:**
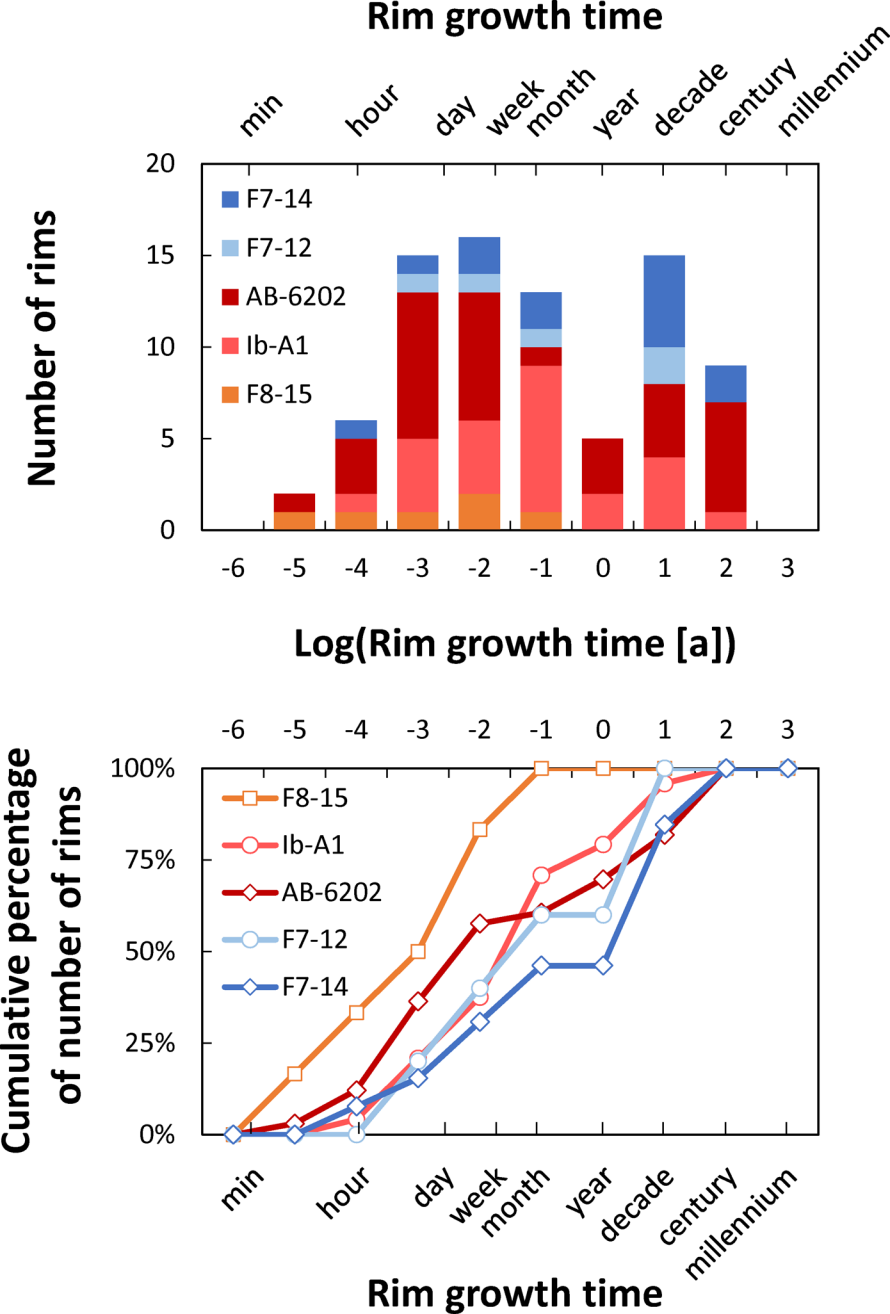
Timescales of rim crystallization for the Bishop Tuff, CA. Top panel shows histogram of calculated times. Bottom panel shows cumulative distributions. Notice that bin sizes are on a logarithmic scale on both plots. Crystals from samples F8-15 (fall unit F8), Ib-A1 (flow unit Ig1Eb), and AB-6202 (flow unit Ig2NW) were imaged at 5 kV (red and orange colors), while those from F7-12 and F7-14 (both from fall unit F7) were imaged at 15 kV (blue colors); see text for details. Distribution has a mode at times of 10^−2^ years (~3 days), and more than 50% of the calculated times are less than 0.1 years (~1 month), particularly so for the crystals imaged at 5 kV.

Rim growth times resulting from our calculations (Fig 5; S1 Table) span from 2*10^−6^ years (~1 min) to 35 years, with distinct modes between 0.001 and 0.01 years (~9 hours to 4 days) and between 1 and 10 years; the median of the entire dataset is 0.01 years (~4 days). All of the samples studied yield minimum rim crystallization times on the order of minutes to hours, and they have broadly similar distributions, including a mode for times less than a year; there is consistent behavior across samples deriving from different stratigraphic units and localities (fall units F7 and F8, and flow unit Ig2E from Chalfant Quarry, and flow unit Ig2NW from Aeolian Buttes). Most samples (all but sample F8-15) also have a mode at longer times (~10 years). Caution is necessary to avoid over-interpreting these results, particularly when analyzing the presence of this mode at longer times, since we present distributions of maximum times, rather than distributions of best estimates. Importantly, more than 70% of rim growth times obtained by us are less than 1 year long. Even if we take a conservative approach and consider the median values derived from our 11 profiles (instead of the minimum), crystallization times are between 1.1*10^−2^ years (~4 days) and 45 years, with a median of 8 years. Overall, our results show that quartz rims primarily grew within a year of eruption, likely with significant growth in the days to months prior to eruption.

### Rim growth rates

**Fig 6:**
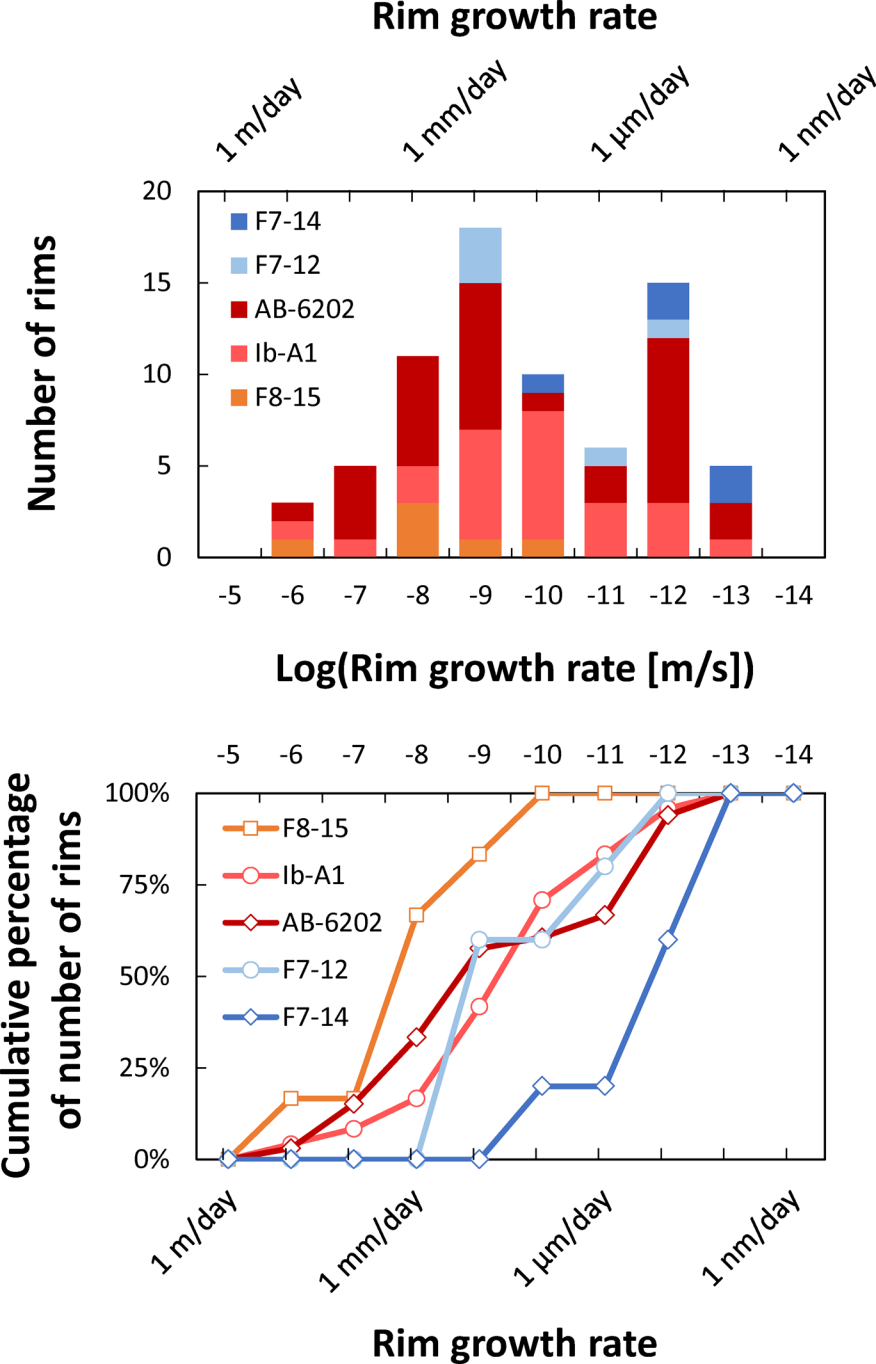
Crystal rim growth rates for the Bishop Tuff, CA. Top panel shows histogram of calculated growth rates. Bottom panel shows cumulative distributions. Notice that bin sizes are on a logarithmic scale on both plots. Distribution has a mode at growth rates of 10^−9^ m/s (~90 μm/day), and more than 50% of the calculated growth rates are faster than 10^−10^ m/s (~9 μm/day).

By combining rim growth times with the widths of the rims measured from the CL images, we can derive average rim growth rates. Measured growth distances vary between 8 and 206 μm (S1 Table), with a median rim width of 41 μm. Resulting growth rates vary between 10^−14^ and 10^−6^ m/s (Fig 6; S1 Table), with a median of 1.1*10^−10^ m/s (~10 μm/day). The resulting distribution is clearly bimodal, with one mode between 10^−10^ and 10^−9^ m/s and another mode between 10^−13^ and 10^−12^ m/s. For growth rates derived from the median of 11 individual profiles, growth rates have a mode between 10^−13^ and 10^−12^ m/s and a median of 1.9*10^−13^ m/s (~16 nm/day). We conclude that, at least for some of the rims we have imaged, crystallization proceeded under conditions that yielded very fast crystal growth rates, on the order of several to tens of micrometers per day; growth rates of hundreds of nanometers per day may have been more typical.

## Discussion

### The origin of crystal rims

The timescales for rim growth derived here place important constraints on the processes that led to the origin of such rims. Since the recognition that temperature can affect the partitioning of Ti into quartz [46], it has become commonplace to assume that bright-CL, high-Ti rims in quartz from volcanic rocks imply a thermal event that may have served as an eruption trigger (e.g. [32]). For many–if not most–supereruption systems, there are significant difficulties with this scenario. Foremost, high-silica magmas saturated in quartz and two feldspars–such as those from the Bishop Tuff system–are nearly-invariant magmas [14]. In such systems, temperature cannot be easily changed, because either one of these phases (i.e. quartz or one of the feldspars) has to be completely consumed, or some other intensive parameter (e.g. pressure) has to change [28, 44]. Additionally, the rims record a crystallization event, which correlates with a nucleation event that gave rise to a microlite population [30], such that special circumstances need to be called upon to promote crystal nucleation and growth of an initially nearly-invariant system under increased temperatures [32, 49]. The timescales we derive here for rim growth pose further challenges for an explanation involving a thermal event. Rim growth is observed in a magmatic system dominated by crystal-poor magma with total volume on the order of 1,000 km^3^ [2, 9]. Whether the Bishop Tuff originated from a single large reservoir with rather independent domains [49] or from separate, independent magma bodies [10] it is difficult to envision a scenario in which injection of new magma could affect such an extensive and discontinuous system on timescales of days to months.

Recognition that pressure also has an effect on Ti partitioning [47]–despite having generated significant controversy [50–53]–provides another possible interpretation for the origin of crystal rims: higher Ti concentrations in the rims could result from syn-eruptive growth during decompression. This would also explain the nucleation event revealed by crystal size distributions [30]. It is important to emphasize that phase relations in quartz-feldspar-bearing systems [54–58] are such that a pressure decrease leads to the displacement of the quartz-feldspar cotectic surface to higher temperatures and higher silica contents; as a result, both quartz and feldspars would remain saturated, and would continue to grow under decreasing pressure. Because supersaturation is increased, growth rates would also increase. Such a scenario explains the timescales and growth rates observed here, as well as the nucleation rates inferred from crystal size distributions [30]. This stands in stark contrast with the case in which temperature is raised, which would lead to undersaturation in one of the felsic phases, would tend to decrease supersaturation, suppressing nucleation and reducing growth rates.

Finally, growth rates also affect Ti incorporation in quartz, with higher concentrations resulting from higher growth rates [53]. We demonstrate here that growth rates were possibly as much as 5 orders of magnitude faster during rim growth, which could be sufficient to increase Ti incorporation in quartz due to kinetic factors [59]–rather than by changes in intensive parameters.

Importantly, both pressure decrease and growth rate increase could act in tandem to promote higher Ti concentration in quartz rims. An alternative view emerges, in which crystal rims record a period of increased growth rate related to syn-eruptive decompression, with no need to appeal to new magma injection or external triggers to explain the onset of the Bishop supereruption.

In this context, rim-growth timescales presented here demonstrate with unprecedented precision the timescales over which the transition from pre-eruptive, heat-loss driven crystallization to syn-eruptive, decompression-driven crystallization takes place: we conclude that syn-eruptive decompression and crystallization initiated most likely within a year of eruption, but with most of the syn-eruptive growth taking place in the days to weeks preceding eruption.

### The lifecycle of giant magma bodies

**Fig 7:**
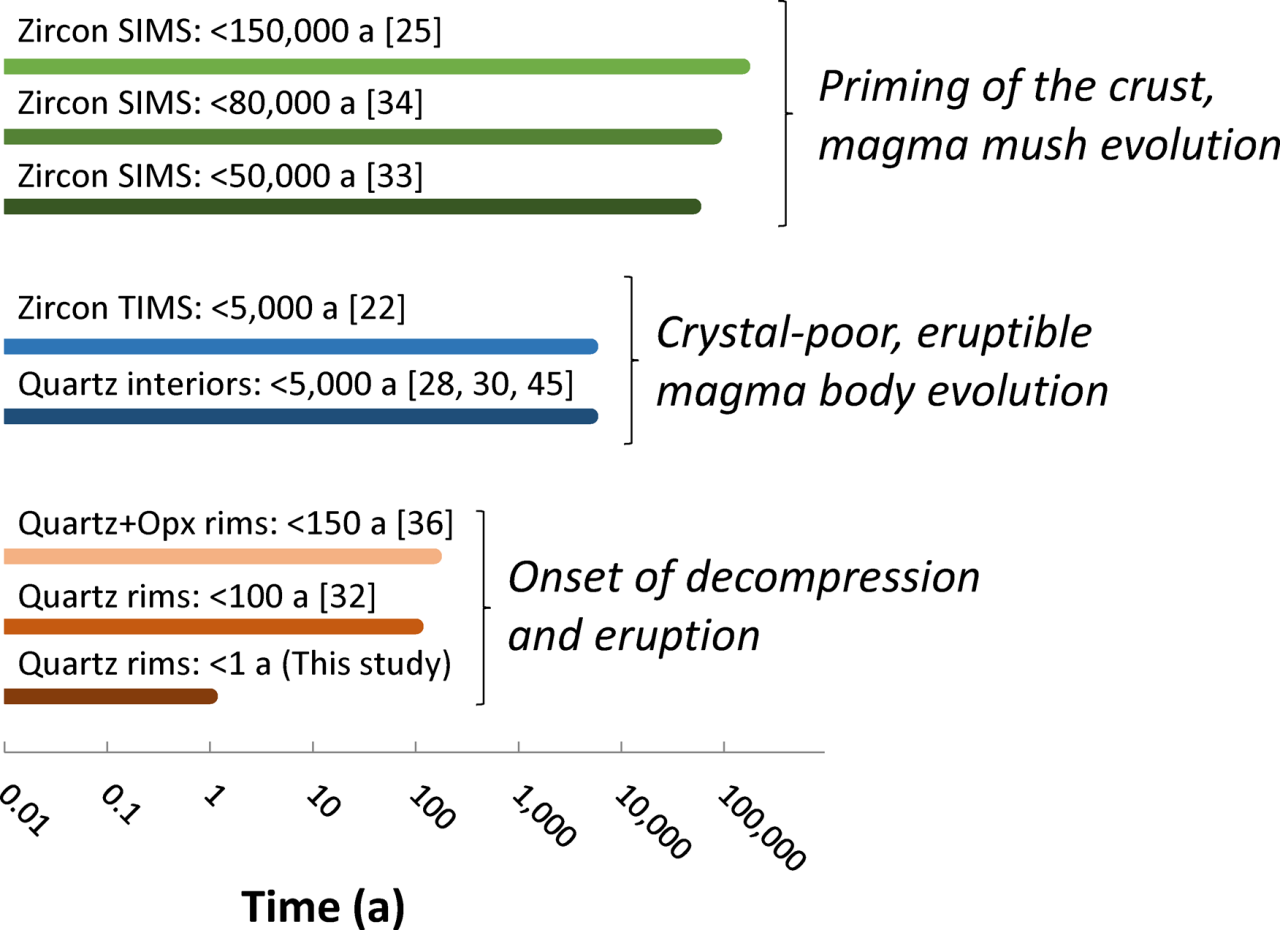
Summary of estimates of the duration of crystallization for the Bishop Tuff. Zircon crystallization times are typically on the order of tens of thousands of years. Quartz interiors reveal crystallization timescales on the order of centuries to a few millennia. Quartz and orthopyroxene (Opx) rims have been previously inferred to have crystallized on decadal to centennial timescales. We show in this study that quartz rims mostly crystallized within a year of eruption, with most growth taking place in the days to weeks prior to eruption. Much of the discrepancy between the timescales results from different minerals, or different zones within the same mineral, recording different processes. Zircon records the evolution of the entire magmatic system, including long stages characterized by high crystal contents; quartz interiors and much of zircon crystallized within centuries to millennia of eruption, revealing the lifespan of crystal-poor, eruptible magma bodies; quartz rims reveal the onset of eruptive decompression. SIMS: secondary ionization mass spectrometry; TIMS: thermal ionization mass spectrometry.

The evolution of giant magma bodies is punctuated by events at several distinct timescales [60] (Fig 7). It is thus useful to put our results in perspective and compare them with other estimates of longevity of the Bishop Tuff magmas.

Zircon ages–both for cores and rims–record crystallization over tens of thousands to hundreds of thousands of years [25, 33, 34]. They record the time necessary to prepare the crust to generate enough melt to produce giant magma bodies. These timescales are likely conditioned by the supply of heat to the crust [61]. Over these timescales, the magmatic system probably waxes and wanes, but–at least in the case of the Bishop Tuff–no significant eruption takes place.

Crystallization times for quartz crystal interiors based on diffusion chronometry [28], melt inclusion faceting [45], and crystal size distributions [30] are on the order of several hundred to a few thousand years, with corresponding growth rates on the order of 10^−13^–10^−14^ m/s. Therefore, the establishment of the melt-rich magma bodies that fed the Bishop eruption most likely took place within a few millennia of eruption. These centennial to millennial growth times reveal the timescales over which giant magma bodies evolve after they form, and they reveal that giant magma bodies are ephemeral features of the Earth’s crust. These timescales are primarily controlled by heat loss to the adjacent crust [28].

Finally, eruptive decompression begins within a year of eruption, and ultimately leads to a supereruption. We speculate that these timescales are a result of the timescales over which crustal deformation takes place prior to wholesale eruption. Our results suggest that decompression and growth probably accelerate over time leading to eruption. If multiple independent magma bodies are present in a region–as it has been proposed for the Bishop Tuff [10] and elsewhere [62, 63]–destabilization of one of these bodies may cause destabilization and eruption of nearby crystal-poor magma bodies, leading to the simultaneous or sequential tapping of a complex mosaic of distinct magma bodies during a single eruption [62] or as part of different but coeval eruptions [63].

## Conclusions

In this study, we present CL and x-ray microprobe data used to characterize the transition between rim and interior zones within quartz crystals from the Bishop Tuff (California). We demonstrate that–as previously suggested–high-CL rims are characterized by higher Ti contents than crystal interiors. We also show that the transition between rim and interior is very sharp, sharper than what can be measured through direct analysis of Ti concentration via the x-ray microprobe. We thus take advantage of the correlation between Ti concentration and CL intensity to better characterize this transition. We use low-energy (5 kV) CL imaging using an SEM, which leads to much higher resolution images due to much smaller interaction volume achieved at these energies, while still retaining enough contrast to properly image the difference in CL intensity between rim and interior.

We use a 1D model to calculate the rim growth times through Ti diffusional relaxation times using data from CL images. We calculate minimum growth rates by combining the growth times with rim thicknesses as measured in the CL images. Maximum rim growth times span from ~1 min to 35 years, with a median of ~4 days. More than 70% of rim growth times are less than 1 year, showing that quartz rims have mostly grown in the days to months prior to eruption. Minimum growth rates have distinct modes between 10^−8^ and 10^−10^ m/s, revealing very fast crystal growth rates, on the order of 100s of nm to 10s of μm per day. Our data show that quartz rims grew well within a year of eruption, with most of the growth taking place in the weeks or days preceding eruption, at rates that are as much as 5 orders of magnitude faster than those characteristic of crystal interiors.

These fast growth rates combined with the presence of a population of microlites that indicate enhanced nucleation suggest that rim growth took place under conditions of high supersaturation. The combination of short timescales and high supersaturation associated with rim growth suggests crystallization during decompression, with no need for changes in crystallization temperature, as commonly assumed. In this context, the growth of quartz rims (and associated growth of microlites) marks the onset of decompression characteristic of the transition from pre-eruptive to syn-eruptive conditions.

The evolution of a giant (supereruption-feeding) magma body is characterized by events taking place at a variety of timescales. Timescales on the order of tens of thousands of years recorded in zircon reveal the time needed to prime the crust in order to generate sufficient eruptible melt. Once established, melt-rich, giant magma bodies are unstable features in the crust, which last for only centuries to a few millennia. We argue that rim crystallization marks the onset of decompression, which takes place within a year from eruption. Most of the rim growth takes place within weeks to days of eruption, suggesting that decompression accelerates over time leading to a supereruption.

## Supporting Information

S1 Table Results from diffusion chronometry. (XLSX)
